# Age of second language acquisition affects nonverbal conflict processing in children: an fMRI study

**DOI:** 10.1002/brb3.246

**Published:** 2014-07-04

**Authors:** Seyede Ghazal Mohades, Esli Struys, Peter Van Schuerbeek, Chris Baeken, Piet Van De Craen, Robert Luypaert

**Affiliations:** 1 Medical Imaging Department (BEFY), Vrije Universiteit Brussel (VUB)Brussels, Belgium; 2 Radiology Department, UZ BrusselBrussels, Belgium; 3 Department of Linguistics, Vrije Universiteit BrusselBrussels, Belgium; 4 Department of Psychiatry, UZ BrusselBrussels, Belgium; 5 Department of Psychiatry and Medical Psychology, Ghent UniversityGhent, Belgium

**Keywords:** Bilingualism, children, conflict, congruency effect, fMRI, Simon, Stroop

## Abstract

**Background:**

In their daily communication, bilinguals switch between two languages, a process that involves the selection of a target language and minimization of interference from a nontarget language. Previous studies have uncovered the neural structure in bilinguals and the activation patterns associated with performing verbal conflict tasks. One question that remains, however is whether this extra verbal switching affects brain function during nonverbal conflict tasks.

**Methods:**

In this study, we have used fMRI to investigate the impact of bilingualism in children performing two nonverbal tasks involving stimulus–stimulus and stimulus–response conflicts. Three groups of 8–11-year-old children – bilinguals from birth (2L1), second language learners (L2L), and a control group of monolinguals (1L1) – were scanned while performing a color Simon and a numerical Stroop task. Reaction times and accuracy were logged.

**Results:**

Compared to monolingual controls, bilingual children showed higher behavioral congruency effect of these tasks, which is matched by the recruitment of brain regions that are generally used in general cognitive control, language processing or to solve language conflict situations in bilinguals (caudate nucleus, posterior cingulate gyrus, STG, precuneus). Further, the activation of these areas was found to be higher in 2L1 compared to L2L.

**Conclusion:**

The coupling of longer reaction times to the recruitment of extra language-related brain areas supports the hypothesis that when dealing with language conflicts the specialization of bilinguals hampers the way they can process with nonverbal conflicts, at least at early stages in life.

## Introduction

There has been a growing interest in the effects of bilingualism on brain function; one focus lies on the study of generic cognitive control skills like inhibition of task-irrelevant features or switching between tasks. In the daily use of their two languages, bilinguals have to continuously resolve interlingual conflicts, possibly leading to conflict-specific brain adaptations. The assumption has been raised that this constant need of solving language conflicts in bilinguals may affect the way in which the bilingual brain deals with general-purpose cognitive control (Abutalebi 2008). In view of this extensive training in solving language conflict situations some researchers have expressed the possibility of a bilingual advantage in conflict resolution across the life span (Bialystok et al. 2004, 2005; Costa et al. 2008). Others have argued against the existence of such an advantage (Morton and Harper 2007; Paap and Greenberg 2013). These contradictory results can possibly be explained by differences in experimental design or the influence of confounding variables, like socioeconomic status or ethnicity (Morton and Harper 2007; Costa et al. 2009).

The inhibition of task-irrelevant information is an important cognitive skill given our limited processing capacity (Klingberg 2000; Johnson et al. 2003). Taxonomies of conflict tasks tapping into inhibitory control reveal a distinction between stimulus–stimulus (S–S) and stimulus–response (S–R) conflict tasks. A stimulus–stimulus (S–S) conflict occurs at the stage of stimulus identification. An operational example is a Stroop task, in which a task-irrelevant feature of the stimulus (for example, the color of a written word or the physical size of a projected digit) may interfere with its task-relevant feature (for example, the meaning of the word or the numerical size of the digit) (MacLeod 1992). Stimulus–response (S–R) conflicts on the other hand occur at the stage of response selection. They arise when task-irrelevant information generates an automatic response that interferes with the required response (Simon and Rudell 1967). An operational example of this is a Simon task, in which a conflict is generated when the location of a stimulus eliciting a given response does not match a position specified in the task instruction (for instance, when the stimulus appears on the right hand side of the screen and the instructions lead to pressing the left button and vice versa). In numerous earlier studies, Simon and Stroop tasks have, respectively been used as practical and classical representations for S–R and S–S conflict types (Simon and Rudell, 1967; Peterson et al. 2002; Egner et al. 2007).

Cognitive processing and conflict resolving are mediated by the executive system. Typical brain regions that modulate such processes are located in the prefrontal areas of the frontal lobe (Alvarez and Emory 2006). Here, the dorsolateral prefrontal cortex (DLPFC) is also associated with response inhibition (conflict processing), working memory, problem solving, and verbal fluency (Lezak 2004; Clark et al. 2008). Besides that the anterior cingulate cortex (ACC) drives cognitive functions and decision making, this area is involved in suppressing irrelevant responses and in processing conflict (Allman et al. 2001). Of note, other brain structures such as the thalamus, hippocampus, and the basal ganglia have also been reported to play role in cognition and conflict processing.

Bialystok found that bilinguals had a behavioral advantage over monolinguals for the Simon task, indicated by shorter reaction times. This advantage was present in three different age groups: young children (5 years), middle-aged adults (30–60 years), and older adults (over 60 years) (Bialystok and Craik 2010). This behavioral advantage was lacking in a group of young adults (20–30 years). The explanation given for these results was that, unless subjects are at the peak of their attention abilities, bilingualism enhances cognitive control processes. By using a Simon task, these results were confirmed by Meuter et al., who reported a better performance for elderly bilinguals (Meuter and Simmond 2007), and by Bialystok and coworkers (Bialystok et al. 2004) who showed that bilingualism reduces age-related increase in distractibility. However, another study using the Simon task in five-year-old bilingual and monolingual children (Morton and Harper 2007) did not reveal a bilingual advantage. This negative result was ascribed by the authors to a better control in their study for differences in socioeconomic status and ethnicity. Until now, all studies have been limited to a Simon task.

Because the two types of conflicts (S–S and S–R) happen at different stages of information processing, they activate the corresponding brain regions in successive time frames (Frühholz et al. 2010). It is well-known that language processing can involve various temporal and spatial layers and stages. This has been attested in studies of, for example, reading proficiency (Price et al. 1999; Price 2000), visual word recognition (Dehaene et al. 2005), and auditory processing (Friederici 2002). The Bilingual Interactive Activation model of language processing (Dijkstra and van Heuven 1998) introduces two distinct systems related to word identification and task decision, respectively (van Heuven and Dijkstra 2010). Conflicts may arise in both systems. Interlingual homographs (i.e., words with the same spelling in two languages, but with a different meaning), for example, generate a conflict at the level of stimulus identification, while a conflict at the level of response selection may appear when one interpretation is linked to a positive response and the other to a negative response.

Some aspects of language-related conflict processing have been studied earlier. van Heuven et al. (2008) have investigated the neural correlates of language conflicts in bilinguals using two tasks. In the first task, participants had to indicate whether a letter string corresponded to a correctly spelled word, leading to conflicts at the stage of stimulus identification. In the second, participants had to indicate whether a given word existed in English, creating conflicts at the stage of response selection. The two tasks were found to activate the left prefrontal cortex in bilinguals which is associated with phonological and semantic processing, but only the response selection conflict task generated activity in the anterior cingulate cortex, basal ganglia, and the supplementary motor area.

Potential language conflict is not restricted to speech comprehension, but may also arise at the level of speech production (Costa et al. 2006). According to Green (1998) bilinguals need to control their language production by inhibiting the nontarget language. When a concept is being worded in one language, associated words in the other language may be activated as well and should be suppressed (Dijkstra 2005).

The ability to suppress impulses and actions is the result of a fundamental mechanism of cognitive control which is known to be served by the right inferior frontal cortex (Forstmann et al. 2008). Some differences have been found between the neural correlates of cognitive control in bilinguals and monolinguals (Henandez et al. 1999; Waldie et al. 2009). For example, in a nonverbal switching task bilinguals were found to activate also the left inferior frontal cortex, part of a network that underlies language control (Garbin et al. 2010). Thus, it seems that handling more than one language affects the location of brain activation related to cognitive control.

In order to test the hypothesis that bilingualism affects general conflict resolution, when there is any kind of conflict between multiple competing presentations, a number of studies have been carried out in which the performance of bilinguals from different age groups in nonlinguistic cognitive conflict tasks was compared to that of monolinguals [(Bialystok et al. 2005; Abutalebi and Green 2007; van Heuven et al. 2008; Costa et al. 2009; Prior and Gollan 2011)].

However, the above-mentioned studies on the impact of bilingualism on general conflict resolution (Bialystok et al. 2005; Abutalebi and Green 2007; Costa et al. 2009) had three important limitations. First of all, only behavioral data (e.g., reaction times, switching costs, and accuracy rates etc.) were collected on the performance of bilingual children (Bialystok et al. 2005; Morton and Harper 2007). Functional magnetic resonance imaging (fMRI) applied in children (Wilke et al. 2003) may improve our understanding of a possible bilingual advantage. To our knowledge, no study has collected neuroimaging data of children with different linguistic skills, performing conflict tasks under fMRI. Second, although fMRI results showing distinct activation patterns depending on the nature of the conflict task were obtained in a general population (adults, language situation unspecified), these results did not include a comparison of the bilingualism-related features of the two types of conflict (S–S and S–R) (Frühholz et al. 2010). Third, in earlier functional and behavioral studies, in bilingual children on conflict resolving abilities, no distinction was made between bilinguals' cognitive processing influenced by age of second language (L2) acquisition, the degree of proficiency, and the degree of exposure to the two languages. Research has shown that notably the age of L2 acquisition has an important effect on differences in the localization of second language activations in the brain (Kim et al. 1997).

The aim of this study is to try and resolve these limitations. First, it provides a direct comparison of the behavioral performance in S–S and S–R conflict tasks between a population of bilingual and monolingual children. Second, it reports the brain activity in this population as recorded by fMRI. Third, to our knowledge, it is the first study to make a distinction between 2L1 and L2L subjects during the performance of S–R an S–S nonverbal conflict tasks.

Bilingualism from birth (2L1) refers to a situation in which children have been concurrently exposed to two languages before the age of two (De Houwer 1996). L2 learning (L2L) is a situation in which a second language is added at a later stage. These two forms of bilingualism may differ in the way the acquired languages interact. For the 2L1s, both languages are balanced and there is little or no separation between their domains of usage. Hence, a high potential for language interference and a high need for inhibiting the nontarget language can be expected. On the other hand, L2Ls have learnt their second language at a later stage in life, presumably leading to a clear functional separation between the first and the second language. One may expect that these differences in language background have an effect on conflict resolution.

The reason to focus on primary school children in this study was the incomplete development of cognitive control in children. As (Piaget 1962) has reported and others have investigated this in detail (McShane 1991; Hurley, May 29, 2012), the cognitive development has four stages: (1) Sensorimotor (0–2 years of life); (2) Preoperational (age 2–7 years); (3) Concrete operations (age 7–11 years); (4) Formal operations (adolescence). At the age of 9 (corresponding to our study), children are already flexible in their mental status and are able to perform concrete mental operations that require considering multiple information simultaneously (http://social.jrank.org, 2013).

Following the results obtained by (Bialystok et al. 2004), we expected to see the differences in conflict resolution performance related to the language background of the participants.

Based on the observed cognitive processing differen-ces between bilinguals and monolinguals (Bialystok, 1999; Festman and Munte, 2012; Linck, et al., 2012; Prior and Gollan 2011), we can conjecture that bilingualism affects the behavioral and functional performance of children's brains in nonverbal conflict resolution tasks (Stimulus-based conflict in e.g., word identification and response-based conflict e.g., in interlingual homographs, respectively Stroop task and Simon task in our study). As bilingualism effects can be expected to be the strongest in simultaneous bilinguals, we expected sequential and simultaneous bilinguals to exhibit different performances in conflict resolution tasks.

## Materials and Methods

### Population

Fifty one right-handed healthy male and female children, aged 96–141 months (mean: 114, SD: 11) and subdivided into three groups (19 bilinguals from birth [2L1], 18 second language learners [L2L], and 14 monolinguals [1L1]) were scanned. All subjects had French or Dutch as first language and the second language of the bilinguals was restricted to Romance or Germanic languages, two branches of the Indo-European language family. The three groups had very similar age and gender distributions (see Table 1). None of the children had any sign of linguistic, neurological or psychiatric disorder and all had normal eyesight.

**Table 1:** Initial group information

Group	Number of subjects	Age (Mean [SD]) [Months]	Gender (F/M)
Bilinguals from birth	19	113 (11)	10/9
L2 learners	18	114 (10)	9/9
Monolinguals	14	115 (12)	7/7

The linguistic background, socioeconomic status, handedness (Edinburgh Handedness Inventory), second language manner of acquisition, and the level of proficiency of all the participants were initially assessed by a detailed questionnaire that was filled out by their parents.

For all bilinguals, frequent use of both languages was reported; 2L1s acquired both languages from birth at home while L2Ls acquired the second language after the age of 3–5 at school. Proficiency was reported by the parents. Only highly proficient children were included in the study.

Verbal auditory discrimination and verbal fluency tests were applied to all subjects in order to assess language reception and production at the semantic level. Listening-comprehension and sentence-construction tests were used to assess these two factors at the syntactic level. Bilinguals underwent these tests in both languages, followed by a bilingual test. In the latter, participants were asked to translate words and sentences from the first language (L1) to the second (L2) and vice versa. They also had to assess the grammatical correctness of the given sentences, possibly containing interference errors from L1 into L2 or vice versa. Children who scored below 50 percent (*n* = 3) on one of these tests were excluded from the experiment.

The study was approved by the Ethics Committee of the University Hospital of Brussels (UZ-Brussel, Belgium) and informed consent was obtained from all parents. As children are naturally inclined to move in the scanner, adequate preparation and a child-friendly atmosphere were provided in order to increase their motivation (Wilke et al. 2003). It was possible to frequently communicate with the children between the scans and they were monitored throughout the experiment via a closed circuit camera system. Parents could follow the proceedings in the scanning room if they wanted.

### Stimuli

The fMRI paradigm consisted of an S–S (numerical Stroop), and an S–R (color Simon) conflict task (Egner et al. 2007). All children were thoroughly instructed and asked to undergo a short demo session outside the scanner in order to avoid possible misunderstanding of the tasks. In addition, at the beginning of each run, a short instruction was projected on the screen to remind the participants of the nature of the task. During the scans, the stimulus information was projected on a screen situated outside the scanner and observed via two mirrors mounted on the head coil. The participants held a response box in each hand and were instructed to press a button on these boxes with their thumbs when appropriate. Reaction times (RT) and accuracy (correct/incorrect) were recorded. The instructions emphasized the importance of both accuracy and speed.

The order and exact timing of stimulus presentation were controlled using E-prime (E-studio Psychology Software Tools http://www.pstnet.com, software release 2.0 Pittsburgh, PA). The timing was generated using efMRI, an fMRI design simulator developed by Chris Rorden (software version 9, see http://www.mricro.com). It was based on a counterbalanced stochastic design, intended to maximize the statistical efficiency while minimizing subject habituation and carry over effects (Henson 2004).

#### Simon task

**Figure 1:**
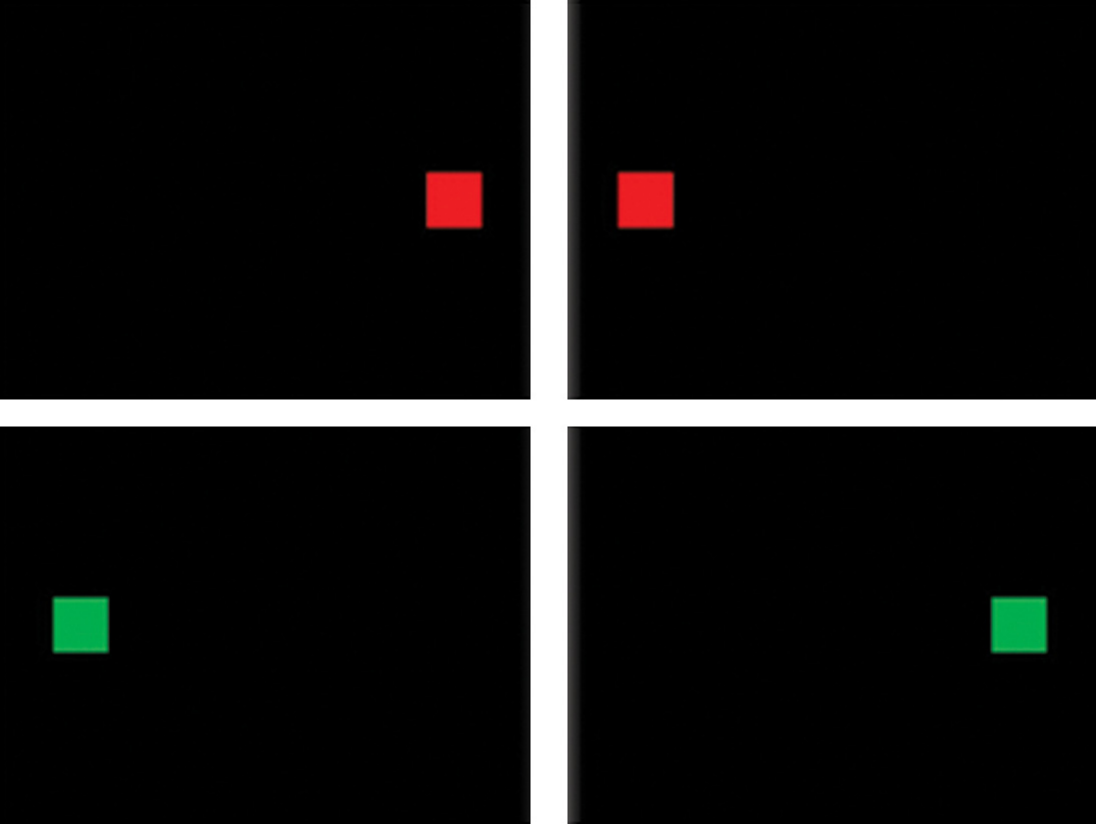
Stimulus presentation for the Simon task. Subjects had to press the right button when a red square appeared and the left when a green square was shown. Top row shows congruent trials, bottom row incongruent trials.

S–R conflicts were studied using an adaptation of the color Simon task (see Fig. 1). Red or green squares projected on a black background were shown to the children. The width of the squares was 10% of the width of the screen. The center of the squares was positioned vertically on the center line of the screen and horizontally at 15% and 85% of its width. Stimuli were classified into two categories: (1) congruent (a red square presented on the right or a green square on the left) and (2) incongruent (a red square shown on the left or a green square on the right). The rapid event-related paradigm lasted 6 min 30 sec and delivered 156 stimuli, 75 of them were congruent, and 81 incongruent. The stimuli were applied with a jittered interstimulus interval (ISI) of 2.2 ± 0.56 sec (maximum ISI = 3.18 s, minimum ISI = 1.19 sec) and a total duration of 6 min and 30 sec. A black background with a centered white fixation cross was projected for 300 ms as the interstimulus rest condition.

The participants had to focus on the color and ignore the position of the figure on the screen. They were asked to press the right button when a red square was shown and the left when a green square appeared on the screen.

#### Stroop task

**Figure 2:**
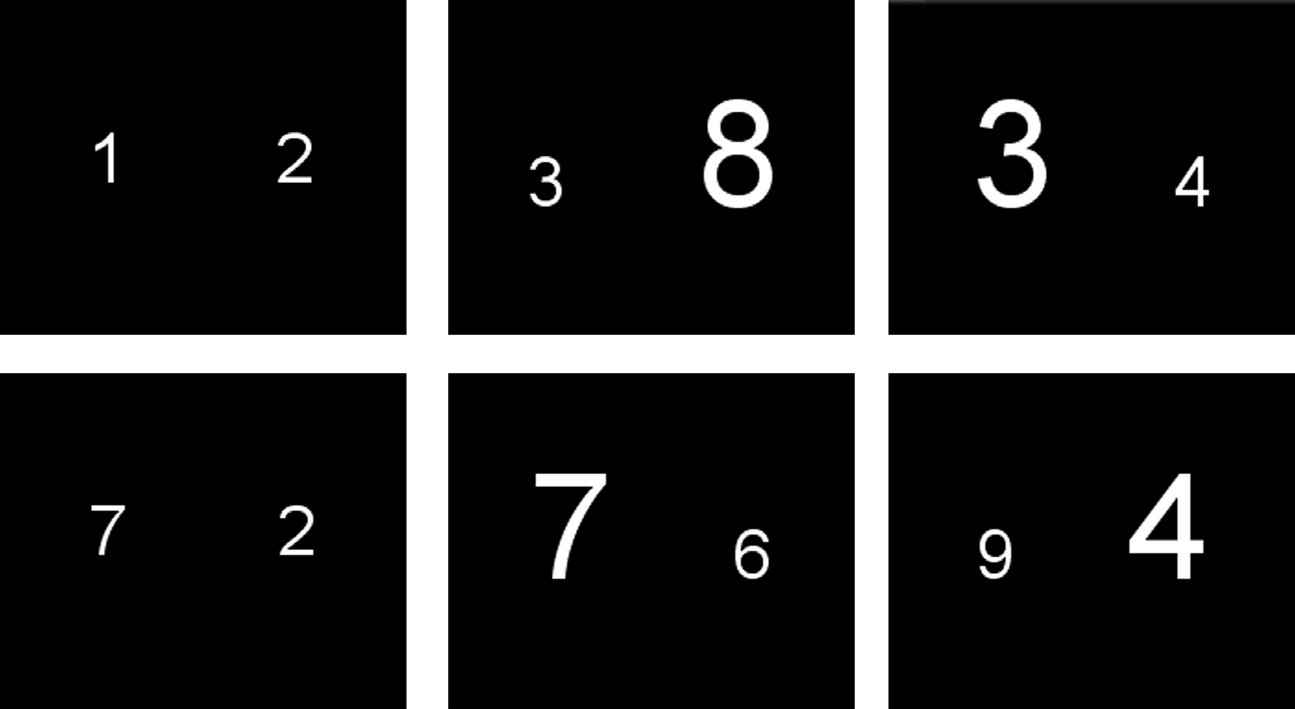
Stimulus presentation for the Stroop task. Subjects had to press the button corresponding to the side where the numerically larger number was shown. Left column: neutral trials; middle column: congruent trials and right column: incongruent trials.

S–S conflicts were assessed using a numerical comparison task. For each trial, two Arabic digits were simultaneously shown to the children and they had to decide which digit was numerically larger, ignoring the physical size of the digits. The stimuli were classified into three categories (Kaufmann et al. 2005): (1) congruent (physical and numerical comparison leading to the same conclusion [e.g., 3 **4**]), (2) incongruent (physical and numerical comparison leading to different conclusions [e.g., **3** 4]), (3) neutral (the stimuli differ only in numerical size [e.g., 3 4]). The neutral trials were added to increase the statistical power. Eight digits were used to create the digit pairs: 1, 2, 3, 4, 6, 7, 8, and 9. The digits were presented in white Arial font on a black background. The two font sizes used were 32 and 58 points. The stimuli were positioned vertically on the center line of the screen and horizontally at 25% and 75% of its width (Fig. 2).

The paradigm included 130 stimuli (43 congruent, 43 incongruent, 44 neutral) and lasted 6 min 30 sec. The stimuli were applied with a jittered ISI (2.7 ± 0.58 sec, maximum ISI = 3.76 sec, minimum ISI = 1.78 sec). At the beginning of each trial a centered white fixation cross on a black background was projected for 300 ms.

### Image acquisition

All scans were performed using a Philips Achieva 3T MR system (software release 2.5) with an eight channel SENSE head coil. BOLD-sensitive T2*-weighted fMRI images were acquired using a spin-echo, echo-planar sequence (EPI) comprising 130 dynamics. Other imaging parameters were: TR/TE=3000 ms/35 ms, FOV = 212 × 230 × 98.5 mm^3^ covering 22 oblique axial 4 mm slices with 0.5 mm gap and matrix size of 104 × 105, total scan duration = 402 sec. Each subject underwent a T1 weighted 3D anatomical scan with following properties: TR/TE = 12 ms/3.75 ms, FOV = 200 × 200 × 200 mm^3^, 100 axial 2 mm slices, 1 × 1 mm^2^ in plane resolution, total scan duration = 6 min and 30 sec.

### Analysis

Seven subjects (four 2L1s, two L2ls, and one 1L1) were discarded from the analysis due to high error rates in one of the tasks or excessive motion (more than 3 mm shift with respect to their first fMRI volume).

#### Behavioral data analysis

The accuracy of the responses (success rate or percentage of correct responses) and the response times (RT) were logged and compared between the groups (ANOVA). This analysis was performed using the Statistical Package for Social Sciences (SPSS 20.0). In addition, in emulation of other authors (Fan et al. 2003; Schulte et al. 2005) the RTs were transformed in “congruency effect” data (Liu and Michigan 2008). This meant that, for each participant, the average RT for the congruent trials of a task was subtracted from the average RT for the incongruent trials yielding a sensitive parameters referred to as “congruency effects”. This congruency effect parameter is used to quantify both the Simon effect (Fan et al. 2003) and the Stroop effect (Hintzman et al. 1972). For the latter the differences “incongruent-neutral” and “neutral-congruent” were also calculated. The congruency effects were compared between the three language groups using an ANOVA. The significance level was set at *P* < 0.05 for a two-tailed test.

#### Image data pre-processing

Image preprocessing and analysis were performed using the SPM8 software (Wellcome Department of Cognitive Neurology, London, UK) running in MATLAB 7.12.

The image files were converted from the Philips PAR/REC format to the Nifti format using r2agui (v2.6; http://www.fil.ion.ucl.ac.uk/spm/ext/). The fMRI volumes of each individual were motion-corrected by realigning them to the first volume of the time series using a rigid-body registration using a least-squares approach. The images were latency-corrected to the 11th slice in each volume. The high-resolution anatomical scan of each subject was coregistered to the realigned functional images.

An age and gender-matched customized pediatric T1-template and tissue priors for gray matter, white matter, and cerebrovascular fluid (CSF) were constructed using the Template-O-Matic (TOM) toolbox (Wilke et al. 2008) (http://dbm.neuro.uni-jena.de/software/tom). TOM creates the template on the basis of the data of 404 healthy 5–18-year-old children acquired in a NIH MRI study (Evans 2006). This template was used instead of the adult brain templates available in SPM and takes into account the developmental changes in size and the shape characteristic of pediatric brains (Wilke et al. 2002).

The anatomical image of each subject was normalized to the aforementioned template using a nonlinear transformation (Friston et al. 1995). The transformation parameters were applied to the corresponding coregistered functional images. The normalized functional images were spatially smoothed using a Gaussian kernel of 8 × 8×8 mm^3^ FWMH.

#### Statistical analysis of the images

##### Fixed effects level

A design matrix based on the information about the conditions and the onsets of the trials was constructed. The time course describing the experimental design was convolved with the canonical hemodynamic response (HRF) function and its time and dispersion derivatives (Hopfinger et al. 2000; Calhoun et al. 2004) in order to model the event-related activity using a 2nd-order Taylor expansion of the response (Friston et al. 1998; Henson 2004). The realignment parameters were included as regressors.

The data were high-pass filtered with a cutoff of 1/128 Hz to eliminate low-frequency noise. Three incongruent–congruent contrast maps (one for each of the convolutions: HRF, time derivative, and dispersion derivative) were calculated for both the Simon and Stroop tasks of each subject. This contrast generalizes the concept of congruency effect introduce earlier. Congruency effects are defined only by subtracting the congruent trials from the incongruent trials for both tasks (Fan et al. 2003; Schulte et al. 2005).

##### Random effects level

A repeated- measures one-way ANOVA of the three contrast maps was used to estimate the main effect of the group for both the Simon and Stroop tasks.

A repeated-measures 3 × 3 ANOVA, was applied for both the Simon and Stroop tasks. The factors in the analysis were “group” (1L1, L2L, and 2L1) and “basis functions” (HRF, time derivative, and dispersion derivative)(Henson and Penny 2003). A combined uncorrected *P*-values of 0.001 and a minimum cluster size of 910 mm^3^ (33 voxels) for Simon task and 740 mm^3^ (28 voxels) for Stroop task was determined using the AlphaSim toolbox (http://afni.nimh.nih.gov/pub/dist/doc/manual/AlphaSim.pdf) (Bennett et al. 2009; Ni et al. 2014).

#### Retrieval of anatomical positions

In the normalization step, an age/gender-matched customized T1-template was constructed using the TOM toolbox. In order to obtain an anatomical label for the activated regions, the activation pattern was overlaid on this pediatric template. Because of the significant differences to be expected between the adult brains depicted in the available automated brain atlases and that of children, these atlases could not be used to look up the anatomical description of the activated regions. The anatomical position of the activities was, therefore, estimated using the graphical information provided by the anatomy textbooks (Scarabino and Salvolini 2006).

## Results

### Behavioral results

The composition of the groups and the behavioral results are summarized in Tables 1 and 2, respectively. The normality of the data sets within the groups was confirmed by the Shapiro–Wilk test. Children with a response error rate exceeding 30% in any condition of the two tasks were excluded (one 1L1 and two 2L1s).

**Table 2:** Response times (in ms) and accuracy scores (in%) for the three groups in the Simon and Stroop tasks

	Simon				Stroop					
		
	Congruent		Incongruent		Congruent		Incongruent		Neutral	
					
Group	RT (SD)	Accuracy (SD)	RT(SD)	Accuracy (SD)	RT (SD)	Accuracy (SD)	RT(SD)	Accuracy (SD)	RT(SD)	Accuracy (SD)
2L1s	658 (98)	96.2 (3.1)	702 (111)	95.4 (3.4)	893 (189)	98.7 (2.1)	1035 (203)	91.4 (4.6)	970 (218)	97.8 (2.6)
L2Ls	692 (90)	93.9 (2.6)	747 (83)	90.7 (6.3)	956 (183)	97.5 (3.1)	1062 (172)	87.5 (7.6)	1002 (168)	98.3 (2.1)
1L1	704 (148)	95.5 (2.8)	735 (149)	94.5 (3.5)	913 (218)	97.2 (1.9)	1007 (244)	88.7 (5.4)	949 (221)	98.1 (2.5)

The mean reaction times (RTs) were based exclusively on the correct responses. The mean RTs of the Simon task were analyzed with a repeated measures ANOVA with Condition type (2 levels) as a within-subjects factor and Group (3 levels) as a between-subjects factor. The results did not reveal a significant main effect of Group, *F*(2, 44) = 1.75, *P* = 0.47, but there was a significant main effect of Condition type, *F*(1, 44) = 46.58, *P* < 0.01, and a significant effect for the Group × Condition type interaction, *F*(2, 44) = 8.75, *P* = 0.04.

However, the main interest of this study, as mentioned before, was the group difference in the congruency effect. A one -way ANOVA for the Congruency effect (RT_(Inc)_ − RT_(cong)_) resulted in a significant group difference, *F*(2, 46) = 2.75, *P* = 0.042 in the Simon task. For post hoc comparisons, see Table 3.

**Table 3:** Post hoc *t*-test results comparing the congruence effects between groups for the two tasks. *P*-values for the relevant *t*-tests are listed

T-values (df)	Simon RT	Stroop RT		
		
*P*-values	Inc-Cong	Inc-Cong	Inc-Neut	Neut-Cong
2L1s > L2Ls	*t*(46) = 5.75 *P* = 0.03	*t*(43) = 2.33 *P* = 0.07	*t*(43) = 10.29 *P* = 0.04	*t*(43) = 9.59 *P* = 0.07
2L1s > 1L1	*t*(46) = 7.92 *P* = 0.05	*t*(43) = 3.24 *P* = 0.045	*t*(43) = 15.23 *P* = 0.05	*t*(43) = 14.41 *P* = 0.03
L2Ls > 1L1	*t*(46) = 13.23 *P* = 0.05	*t*(43) = 1.08 *P* = 0.07	*t*(43) = 5.70 *P* = 0.05	*t*(43) = 5.52 *P* = 0.03

At *P* < 0.05 significance level, all comparisons reach at least marginal significance.

**Figure 3:**
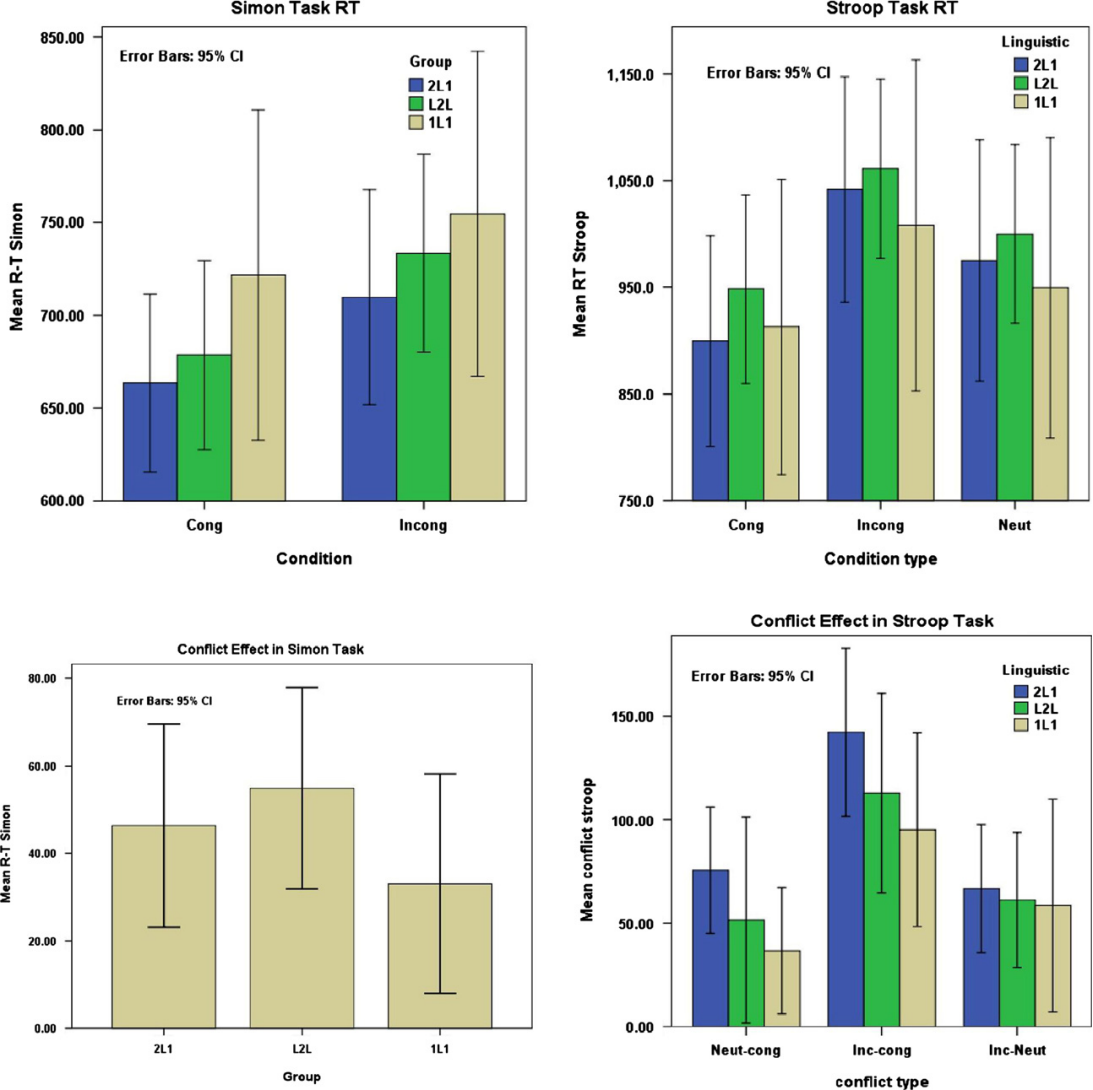
Reaction times and congruity effect on reaction times for Simon and Stroop tasks and for different conditions.

For the RTs of the Stroop task, a repeated measures ANOVA (again with Condition type as a within-subjects factor, and Group as a between-subject factor) did not reveal a significant main effect of Group, *F*(2, 41) = 0.64, *P* = 0.80. However, a main effect of Condition type was found *F*(2, 41) = 55.51, *P* < 0.01, as well as a significant effect for the Group × Condition type interaction, *F*(4, 41) = 8,65, *P* = 0.02. The ANOVAs for the three comparisons of the Congruency measures [Inc-Cong,Neut-Cong, Inc-Neut] for the Stroop task revealed significant group differences as follows: Inc-Cong *F*(2,43) = 12.27, *P* = 0.04, Neut-Cong, *F*(2,43) = 10.92, *P* = 0.04, and Inc-Neut, *F*(2, 43) = 5.7, *P* = 0.03. Bonferroni corrected post hoc *t*-tests for each of these comparisons showed significantly higher congruency effects in bilinguals compared to monolinguals (see Table 3 and Fig. 3). In the three comparisons for the Stroop task, the Congruency measures were largest in the 2L1s, followed by the L2ls and 1L1.

In the Simon task, the average accuracy over the groups was 95.0% (SD: 2.9; range: 88.0–100.0%) for congruent trials and 93.1% (SD: 5.5; range: 77.7–100%) for incongruent trials. For the Stroop task, it was 97.9% (SD: 2.5; range: 86.3–100%) for congruent trials, 89.3% (SD: 6.2; range: 72.0–97.6%) for incongruent trials, and 97.8% (SD: 2.9; range: 86.3–100%) for neutral trials (see Figure S2).

As for the RTs, the mean accuracy rates were analyzed with a repeated measures ANOVA with Condition Type as the within-subjects factor, and Group as the between-subjects factor. For the Simon Task, this revealed a significant main effect of Group, *F*(2, 44) = 5.76, *P* < 0.01, and also a significant main effect of Condition type, *F*(1, 44) = 5,64, *P* = 0.02. No effect was found for the Group × Condition type interaction, *F*(2, 44) = 0.853, *P* = 0.43. Bonferroni-corrected post hoc *t*-tests revealed a significant difference between 1L1s > L2Ls (*P* = 0.02), and also between 2L1s > L2Ls (*P* < 0.01). Figure S2 illustrates the mean accuracy rates for the three groups in both tasks.

For the Stroop task, a highly significant main effect was found for Condition type, *F*(2, 41) = 73.36, *P* < 0.01, but not for Group, *F*(2, 41) = 0.55, *P* = 0.58, nor the interaction between Condition type and Group, *F*(4, 41) = 1.02, *P* = 0.39.

### fMRI results

For individual group activities on both tasks see Figure S1.

#### Between-group comparison for the incongruent- congruent contrast in the Simon task

The 3 × 3 ANOVA revealed a significant main effect of the linguistic group in the Simon task in the inferior frontal gyrus (IFG) (*F*_(2,108)_ = 10.32, *P* < 0.01), caudate nucleus (*F*_(2,108)_ = 8.90), *P* < 0.01), superior temporal gyrus (STG) (*F*_(2,108)_ = 8.78, *P* < 0.01), cingulate gyrus (*F*_(2,108)_ = 8.01, *P* < 0.01), middle temporal gyrus (*F*_(2,108)_ = 7.84, *P <* 0.01), middle frontal gyrus (*F*_(2,108)_ = 7.51, *P <* 0.01).

**Figure 4:**
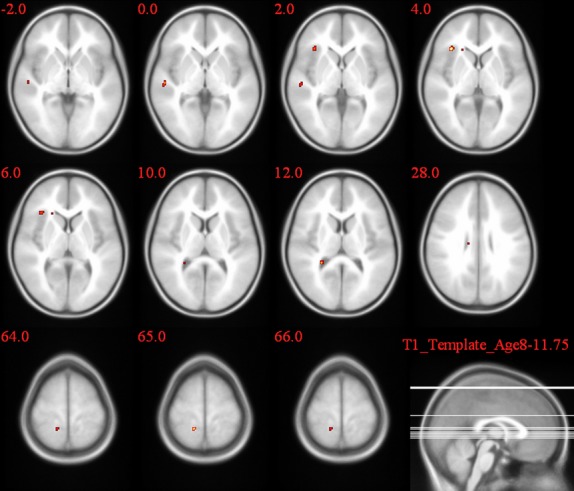
2L1 versus L2L group comparison of the activation pattern for the incongruent–congruent contrast in the Simon task.

**Figure 5:**
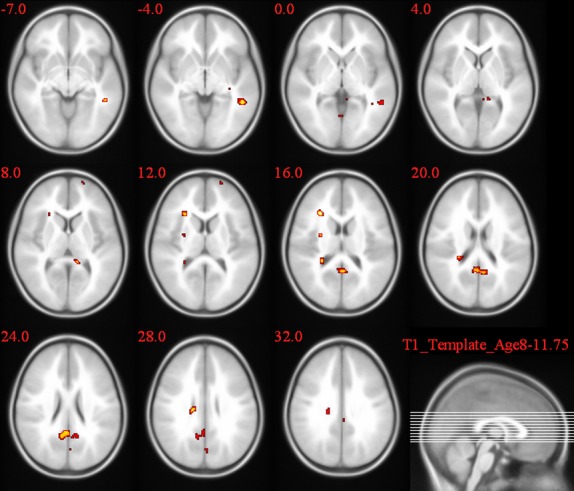
L2L versus 1L1 group comparison of the activation pattern for the incongruent–congruent contrast in the Simon task.

**Figure 6:**
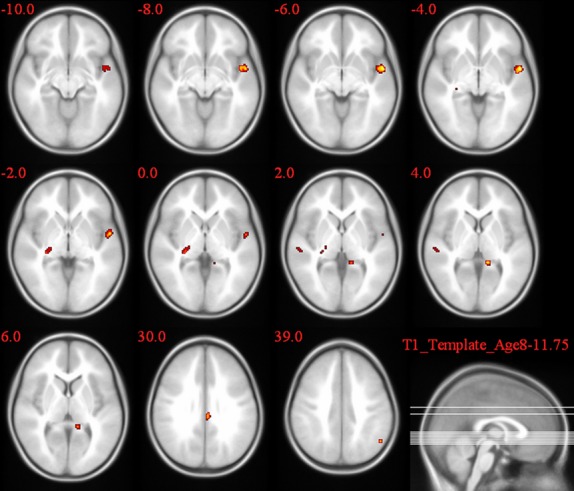
2L1 versus 1L1 group comparison of the activation pattern for the incongruent–congruent contrast in the Simon task.

Figures 4–6 and Table 4 summarize the results of post hoc *t*-test comparisons of the group analysis for the Simon task.

**Table 4:** Post hoc test results revealing regions with significant congruence-effect differences between groups while doing the Simon task (*this comparsion did not reach the significance level)

Simon task incongruent–congruent contrast				

	Side	Brain region	Cluster size	Peak *T* value
2L1s > L2Ls	R	Inferior frontal gyrus	10*	7.22
L2Ls > 1L1	R	Posterior cingulate	86	7.1
				6.42
	R	Middle frontal gyrus	69	7.44
	R	Caudate body	35	8.11
	L	Superior temporal gyrus	41	7.87
	L	Posterior cingulate	140	6.18
2L1s > 1L1	R	Middle frontal gyrus	41	6.65
	R	Middle Temporal gyrus	113	6.36
	R	Precuneus	79	4.05
	L	Superior temporal gyrus		10.65

In group level analysis repeated measures ANOVA reveals enhanced activation while doing the Simon task observed for congruity effect (Incongruent trials–congruent trials).

A number of brain regions showed significantly greater incongruent versus congruent contrast in bilingual participants compared to monolinguals. The activation pattern expected for stimulus–response conflict was confirmed in all the three comparisons. The superior temporal gyrus (STG) exhibited a significantly different congruency effect in all the three comparisons and showed a bilaterally increased activation in 2L1s compared to 1L1s.

A significantly larger congruency effect was observed in 2L1s compared to L2Ls in the caudate body (*T* = 5.92). When comparing L2L and 1L1, following brain regions showed a higher congruency effect: caudate body (*T* = 8.11), left and right post cingulate gyrus (*T* = 7.1, *T* = 7.13), STG (*T* = 7.87), and middle frontal gyrus (*T* = 7.44). 2L1s compared to 1L1 showed a significantly higher congruency effect in the STG (*T* = 10.65), posterior cingulate gyrus (*T* = 8.32), cingulate gyrus (*T* = 7.07), thalamus (*T* = 7.07), Middle frontal gyrus (*T* = 6.65), middle temporal gyrus (*T* = 6.36), and precuneus (*T* = 4.05).

#### Between-group comparison for the incongruent– congruent contrast in the Stroop task

The numerical Stroop task produced a significant congruency effect differences in multiple areas in bilingual brains compared to monolinguals.

The 3 × 3 ANOVA yielded a significant main effect of the group factor in the caudate head (*F*_(2,108)_ = 12.77, *P* < 0.01), cingulate gyrus (*F*_(2,108)_ = 10.25, *P* < 0.01), and middle temporal gyrus (*F*_(2,108)_ = 8.83, *P* < 0.01).

**Figure 7:**
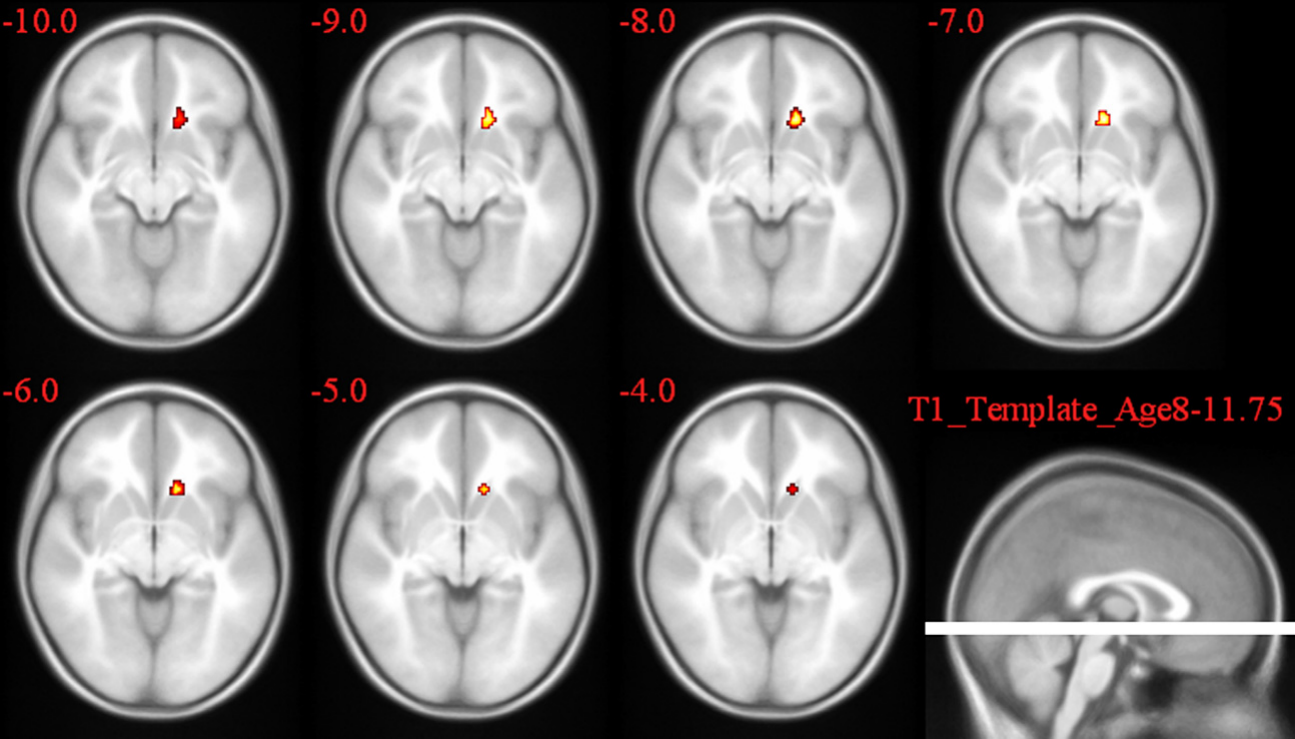
2L1 versus L2L group comparison of the activation pattern for the incongruent–congruent contrast in the Stroop task.

**Figure 8:**
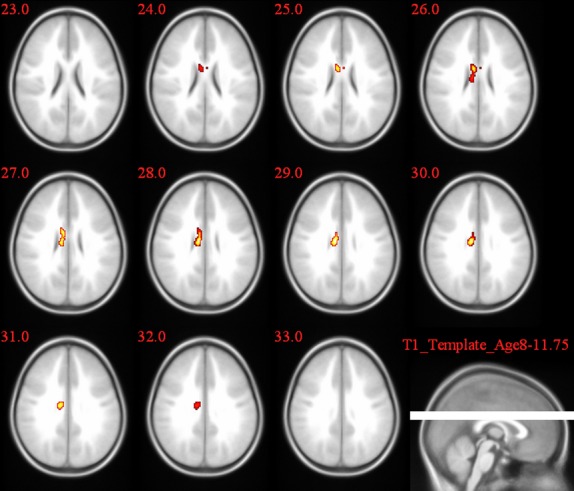
L2L versus 1L1 group comparison of the activation pattern for the incongruent–congruent contrast in the Stroop task.

**Figure 9:**
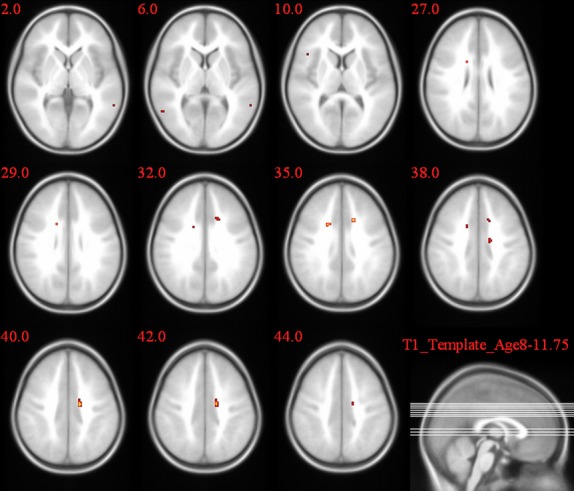
2L1 versus 1L1 group comparison of the activation pattern for the incongruent–congruent contrast in the Stroop task.

In the post hoc *t*-test comparisons, as seen in Table 5 and Figures 7–9, the cingulate gyrus showed a bilateral increased congruency effect in bilinguals (both 2L1s and L2Ls) compared to monolinguals (*T* = 8.01 and *T* = 9.08, respectively). 2L1s in comparison to L2Ls pointed to a higher congruency effect in the caudate head (*T* = 9.67).

**Table 5:** Post hoc test results revealing regions with significant congruence-effect differences between groups while doing the Stroop task (*this comparsion did not reach the significance level)

Stroop task incongruent–congruent contrast				

	Side	Brain region	Cluster size	Peak *T* value
2L1s > L2Ls	L	Caudate head	31	9.67
L2Ls > 1L1	R	Cingulate gyrus	76	9.08 7.83
	L	Cingulate gyrus		6.27
2L1s > 1L1	L	Cingulate gyrus	14*	8.01

In group level analysis repeated measures ANOVA reveals enhanced activation while doing the Stroop task observed for congruity effect (Incongruent trials–congruent trials).

## Discussion

This study has collected behavioral and neuroimaging data for stimulus–stimulus (numeric Stroop task) and stimulus–response (Simon task) conflict tasks. The subjects consisted of bilinguals from birth (2L1), L2 learners (L2L), and monolingual (1L1) children. This group composition aimed to study if different ways of managing languages could affect nonverbal conflict resolution during a crucial period of human brain development. The 2L1s acquired their two languages concurrently in early childhood, using them interchangeably for the same communicative functions. L2Ls acquired their second language after the first, in an educational setting, between the age of three and five, resulting in an operative separation between the languages.

We aimed to monitor the impact of handling more than one language and also of the age of acquisition (AOA) of the second language on the cognitive skill of children which is still under development.

### Behavioral results

In this study, we have controlled for socioeconomic status and ethnicity. Overall, reaction times for the different trial types and accuracy scores showed similar results for all groups. As the reaction times and accuracy may be affected by confounding variables such as anxiety, fatigue, stress, experience on computer games, illness, distraction, etc., we have quantified the congruency effect by subtracting the reaction times for congruent trials from the RT for incongruent trials(Fan et al. 2003; Schulte et al. 2005). [In the Stroop task, two other quantities were also calculated Incongruent-Neutral and Neutral-Congruent see Fig. 3.] Significant differences between all groups in both tasks were found for this new quantity: bilinguals showed higher congruency effects than monolinguals. In the Stroop task, the size of the congruency effects appeared to be related to the degree of exposure to language conflict situations. 2L1s showed higher congruency effects, possibly because the operative overlap of their languages creates more potential for language conflict. In the Simon task, the opposite pattern was seen in the bilingual groups, with the L2Ls showing higher congruency effects. It is unclear why L2Ls show higher congruency effects in an S–S conflict task, given the fact that they have to deal with less conflict in managing the languages they use.

Our results are not in line with the presence of a behavioral advantage of bilingualism throughout life. This absence of an advantage (or even the existence of a disadvantage) at some stages is, however not surprising. It was reported earlier that the many language conflicts encountered by bilinguals may slow down lexical access (Gollan and Kroll 2001). In a review paper, Hichey and Klein, have assessed the bilingual advantage in conflict resolution using nonlinguistic inhibitory tasks (Hilchey and Klein 2011). They concluded that while a bilingual advantage has been seen in a few cases, it is subject to many factors and cannot be generalized. It is possible that the reduced performance in nonverbal conflict processing observed here in bilingual children is only temporary and that they catch up with their monolingual peers at a later age. To confirm this hypothesis, further studies are required in tracing the development of conflict processing in bilingual children and young adults in an extended longitudinal research design.

### fMRI results

In order to investigate the differences in brain activities in the three study groups, fMRI data were collected during the performance of the two conflict tasks. To our knowledge, this study was the first to compare brain activity during an S–S and an S–R task between groups of monolingual and bilingual children. Using magneto-encephalography, one earlier study in adults showed that behavioral differences between monolinguals and bilinguals during Simon task performance can be linked to differences in brain activity (Bialystok et al. 2005). A comparable effect of bilingualism on the locus of brain activity during a nonlinguistic cognitive control task was seen in a recent fMRI study (Garbin et al. 2010): during the execution of a nonverbal switching task, bilinguals activated left hemispheric frontal brain regions responsible for language control, whereas monolinguals showed a predominantly right hemispheric involvement. These neuroimaging results seem to corroborate the idea that daily training in language inhibition influences the brain region recruitment in bilinguals while solving nonverbal conflict situations, even when no behavioral differences are observed.

Another recent study, outside the language context in an adult general public cohort, showed differences in cerebral activation patterns between two types of conflict tasks similar to ours (Frühholz et al. 2010): S–S conflicts activated the anterior cingulate cortex (ACC), with underlying source activity in the inferior frontal cortex, whereas stimulus–response conflicts produced distinct activity in the posterior cingulate cortex (PCC), with underlying source activation in the superior parietal cortex. The anterior cingulate cortex (ACC) is involved in error detection and conflict monitoring (Carter et al. 1998; Bush et al. 2000; Xue et al. 2008). In conflict monitoring hypothesis, Botvinick et al. proposed that ACC would monitor competitions between conflicting representations regardless of correct or false responses (Botvinick et al. 2004).

On the other hand, in an arrow-word Stroop task, Roelofs et al. have demonstrated that ACC activity can be independent of response conflict, challenging the conflict-monitoring view of ACC function. However, they reported more activation for neutral than for congruent stimuli in the absence of response conflict, confirming the involvement of the ACC in conflict processing (Roelofs et al. 2006). The role of the ACC is to report potential conflict, regardless of its nature, to the frontal cortex where the actual process of conflict resolution takes place. Together with the left inferior frontal gyrus, the left striatum, and the left inferior parietal lobe are part of a neural network in charge of controlling language use in multilingual speakers (Abutalebi et al. 2007).

In our study, differences between the two bilingual groups and the monolinguals were only found in the bilateral cingulate cortex for S–S conflict resolution. The cingulate cortex is reliably activated in different tasks involving language use monitoring in bilingual speakers. These tasks entail both language production, such as word translation (Price et al. 1999) and switching into the less-proficient language (Wang et al., 2007), and language reception, such as an auditory perception of language switches during comprehension of narratives (Abutalebi and Green 2007). The anterior part of the cingulate cortex is also involved in managing language use when bilinguals process identically spelled words with different meanings (van Heuven et al. 2008).The linguistic tasks reported in the above-mentioned studies include both language control conflict resolution stages as they cover both language identification and language production. Our findings report the activation of cingulate gyrus in both S–R and S–S conflict monitoring and affirm our hypothesis that encountering language conflicts in daily life affects general cognitive control processes.

In our study, which compared the contrast incongruent–congruent for the two conflict tasks between bilingual children and monolingual controls, we have found extra activated regions in the bilinguals.

One of the activated regions was the caudate nucleus, which is known to be active during learning and linking stimuli and responses (Seger and Cincotta 2005). Caudate nucleus activation was found in L2L group when they were compared to 1L1s during the performance of Simon task, and in the 2L1s when they are compared to the L2L group during the Stroop task.

Based on the previous findings concerning the involvement of the caudate head in inhibition tasks (Shadmehr and Holcomb 1999; Ray Li et al. 2008) and their implication for language switching in bilinguals listening to a narrative (Abutalebi et al. 2007), it has been stated that the caudate nucleus controls both verbal and nonverbal types of conflicts (Bialystok et al. 2009).

Further, in a study investigating language control in bilingual brains Crinion et al. observed left caudate activation in monitoring and controlling the language in use (Crinion et al. 2006). In their study, in which sequential word pairs were presented in German or English, they showed that semantically related words were associated with a reduced activation in the left caudate when prime (1st word) and target (2nd word) were in the same language, but not when they were in different languages. In another study, Schouppe et al. (2014) reported activation in the right and left caudate nucleus in high-conflict choices. This may imply that stimulus-based general conflicts lead to more activation in 2L1s than in L2L children. This conflict type may occur in semantic-priming tasks found in the word identification system (van Heuven et al. 2008).

In view of the study by van Heuven et al. (2008), demonstrating that response-base language conflicts raise activations in the caudate nucleus, and the added activation we have observed in L2Ls compared to 1L1 children in the caudate during the Simon task, we may confirms our hypothesis that nonverbal conflicts in bilinguals give rise to activation in language conflict processing areas in the brain.

Another added region is the posterior cingulate gyrus which showed increased activation in most of the comparisons in this study (see Tables S2 and S3), and is associated with the task-related role of working memory or retrieval processes (Sakai et al. 1998; Tracy et al. 2003). The superior temporal gyrus (STG) was also found to be additionally activated in the group comparisons of the Simon task. The STG is responsible for auditory processing (Bigler et al. 2007) and processing species-specific vocalizations (Karnath 2001).

The comparison between 2L1s and 1L1s during the performance of Simon task showed an increased activation in precuneus in 2L1s, this area is known to play a role in high-order cognitive tasks (Cavanna and Trimble 2006), it has been also reported to be involved in recollection of words (Krause et al. 1999) and integrated in semantic network (Jessen et al. 1999).

One possible explanation for these findings may be that brain regions involved in solving language conflict situations or general language processing, are automatically activated when bilinguals carry out cognitive control tasks. Bilinguals from birth who have to manage their language systems more extensively than L2 learners, as a consequence show more activity in language control regions such as the caudate nucleus (Hernandez et al. 2000; Lehtonen et al. 2005; Crinion et al. 2006; Abutalebi et al. 2008; Tan et al. 2011). Similar differences in all above-mentioned regions were observed between the two bilingual groups and the monolinguals.

The behavioral and functional differences between the two bilingual groups (see Figs. 3,4,7) confirms our primary hypothesis that not only handling more than one language, but also the age and manner of acquiring the second language impact the general cognitive process in the brain of children.

Nevertheless, a limitation of our current study is the absence of language control tasks. Hence, a direct comparison between our findings on nonverbal conflict processing and language control in the brain of children could not be made.

Another limitation of our study is the slight difference in sample sizes between the groups. Even though we controlled for language use, it was impossible to exclude children who had minimal exposure to a second language from our 1L1sample. The reason for this is that, by the age of ten, almost all children in the multilingual Brussels Region have to some extent been exposed to other languages than their mother tongue. Future studies with larger and more equal sample sizes are thus needed to confirm our data. The differences between monolinguals with a little exposure to another language and various types of bilinguals we have found here may show up even more in comparisons involving monolinguals from regions that are less linguistically heterogeneous than Belgium.

#### Role of the age of acquiring L2

Although it had been well-established that conflict processing is different in bilinguals and monolinguals, a question that remained unanswered is whether the age of exposure to L2 affects the cognitive impact of bilingualism in children. So far, all studies on bilingual children include only 2L1s (Bialystok et al. 2004; Carlson and Meltzoff 2008) and research on bilingual cognitive-controlled-processing seems to have overlooked the possible impact of the age of L2 acquisition. Including 2L1s and L2Ls in our study allows a novel comparison between bilingual children that differ on the age of L2 acquisition. Our findings provide preliminary evidence that the age of being exposed to L2 does indeed influence brain activation during nonverbal conflict tasks. In particular, increased activation in the caudate head during Stroop tasks in 2L1s compared to L2L suggests differential performance in high-conflict situations in 2L1s. This level of conflict in bilinguals arises at the stage of word identification and was shown to be different between sequential and simultaneous bilinguals of primary-school age (van Heuven et al. 2008).

Moreover, during the Simon task (response-based conflict) we could establish important differences between the two groups of bilinguals in terms of their relationship with 1L1s. In L2Ls compared to 1L1s, additional activation spots were found in the caudate body and posterior cingulate. Both regions have been reported to be activated during the response-based language conflicts (van Heuven et al. 2008) and our finding may imply that L2Ls compared to monolinguals recruit additional language conflict processing regions in the brain during nonverbal conflict tasks. These results, together with the behavioral difference between the two bilingual groups noted in this study, provide evidence for the impact of age of L2 acquisition on primary school children's cognitive skills.

## Conclusion

This study provides first evidence of the effect of language background on two types of conflict resolution in the brains of bilingual children. In bilingual children compared to monolingual controls matched for age, gender, ethnicity, and socioeconomic background, the behavioral data showed higher congruency effects: reaction times were found to be lengthened and accuracy to be decreased by general cognitive conflict. Although this finding contradicts earlier studies which pointed at a bilingual advantage in conflict resolution, our neuroimaging data also showed a more extensive conflict-related brain activity in bilingual groups in brain regions typically related to language control. The higher behavioral congruency effect is thus matched by the recruitment of brain regions that are generally used by bilinguals to solve language conflict situations. This coupling of a behavioral disadvantage to the recruitment of extra, language-related, brain areas supports the hypothesis that the specialization of bilinguals in dealing with language conflicts hampers the way they can deal with nonverbal conflict, at least at early stages in life.

The results obtained here are contradictory to the common hypothesis of a bilingual advantage in adults, reported in literature (Morton and Harper 2007) and pave the way for the assumption that the conflict processing mechanism may be different in bilingual children and adults.

## Supporting Information

**Figure S1.** The congruency effect [brain activity for incongruent–congruent trials] in each group for the Simon and Stroop tasks.

**Figure S2.** Accuracy rates for Simon and Stroop tasks and for different conditions.
