# Supplementary figures and images for: Age of second language acquisition affects nonverbal conflict processing in children: an fMRI study
Source: Brain Behav. 2014 Jul 4;4(5):626–42. doi: 10.1002/brb3.246 (PMC4107382; doi:10.1002/brb3.246)

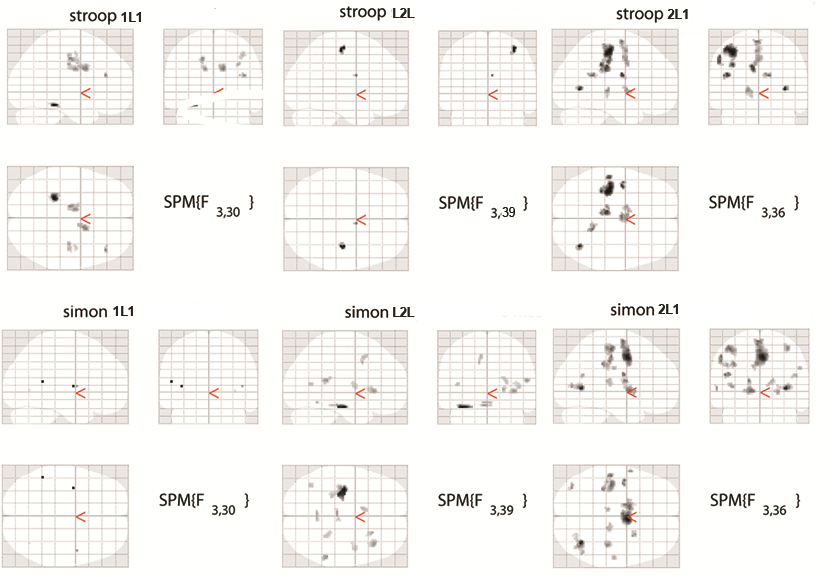

Supplement: Supplementary file 1 — Figure S1. The congruency effect [brain activity for incongruent–congruent trials] in each group for the Simon and Stroop tasks. [file brb30004-0626-SD1.tif]
